# Monitoring insecticide resistance of adult and larval *Aedes aegypti* (Diptera: Culicidae) in Phnom Penh, Cambodia

**DOI:** 10.1186/s13071-022-05156-3

**Published:** 2022-01-31

**Authors:** Sebastien Boyer, Pierre-Olivier Maquart, Kalyan Chhuoy, Kimhuor Suor, Moeun Chhum, Kimly Heng, Sokkeang Leng, Didier Fontenille, Sebastien Marcombe

**Affiliations:** 1 Medical and Veterinary Entomology Unit, Institut Pasteur du Cambodge, 5 Boulevard Monivong, Phnom Penh, Cambodia; 2 International Pasteur Institute Network, Institut Pasteur, Paris, France; 3 MIVEGEC, Université de Montpellier, CNRS, IRD (Institut de Recherche Pour Le Développement), 911 Avenue Agropolis, 34394 Montpellier, France; 4 Medical Entomology Unit, Ministry of Health, Institut Pasteur du Laos, Vientiane, Lao People’s Democratic Republic

## Abstract

**Background:**

Dengue fever is a major public health concern in Cambodia, with thousands of cases every year in urban, suburban and rural areas of the country. The main vector of dengue fever in Cambodia is *Aedes aegypti*. The organophosphate larvicide temephos and adulticides belonging to the pyrethroid family have been widely used for decades by public health authorities to fight dengue vectors, but resistance of *Ae. aegypti* to these insecticides has been previously described for Cambodia.

**Methods:**

In order to adapt the vector control strategy presently used in Cambodia, we tested 14 adulticides belonging to the carbamate, organochlorine, organophosphate, and pyrethroid insecticide families and three larvicides [temephos, spinosad and *Bacillus thuringiensis* ser. *israelensis* (Bti)] belonging to three different insecticide families (organophosphates, spinosyns and entomopathogenic bacteria). The standard procedures used here to test the adults and larvae of an *Ae. aegypti* population from Phnom Penh followed World Health Organization guidelines.

**Results:**

For adults, high mortality rates were observed with carbamate, organophosphate and organochlorine (with the exception of dichlorodiphenyltrichloroethane) insecticides (i.e. between 87.6 and 100%), while low mortality rates were observed with all of the tested pyrethroid insecticides (i.e. between 1 and 35%). For larvae, no resistance against Bti was detected [resistance ratio (RR_90_ < 1.6)], but moderate resistance was observed for temephos and spinosad (RR_90_ < 5.6).

**Conclusions:**

The results of this study indicate that (i) Bti should be considered a serious alternative to temephos for the control of *Ae. aegypti* larvae; and (ii) the carbamate adulticides propoxur and bendiocarb should be employed instead of the widely used pyrethroid insecticides for the control of adult *Ae. aegypti* on land under mosaic farming and crop rotation in Cambodia, as the insects were found to be resistant to the latter types of insecticide. Research focusing on insecticide resistance and innovative and effective vector control strategies should be undertaken as a public health priority in Cambodia.

**Graphical abstract:**

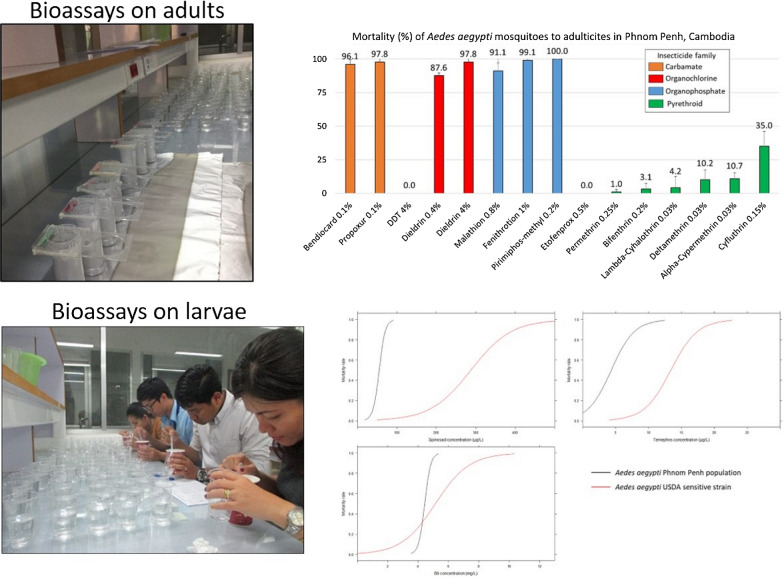

## Background

Dengue fever is a major public health concern as it causes thousands of deaths every year in urban, suburban and rural tropical and sub-tropical areas [1]. Dengue virus, like Zika virus and chikungunya virus, is mainly transmitted by *Aedes aegypti* mosquitoes [2, 3]. In Cambodia, the Ministry of Health (MoH) has been monitoring the incidence of dengue every week since the 1995 outbreak of this virus, which caused more than 400 deaths [4, 5]. The most recent dengue epidemics in Cambodia were in 2007 (39,618 cases, 396 deaths), 2012 (42,362 cases, 189 deaths) and 2019 (68,597 cases, 48 deaths) (MoH, Phnom Penh, Cambodia). In 2018 and 2019, the capital of Cambodia, Phnom Penh, was affected by this disease as never before, with 9,445 and 9,298 cases, respectively [6]. The last arbovirus outbreak in Cambodia was the chikungunya epidemic in 2020, with a total of 1,722 cases throughout the 25 provinces of the country (Duong Veasna, personal communication).

*Aedes aegypti* is distributed throughout Phnom Penh [6] and in the Cambodian countryside [7–9], sometimes in sympatry with *Aedes albopictus*, another important arbovirus vector [6]. Hence, vector control targeting *Ae. aegypti* populations may have a wider public health impact. Temephos (Abate®) has been the most broadly used insecticide in Cambodia since 1992 for vector control targeting larval stages of *Ae. aegypti* [10]. In Southeast Asia, adult* Ae. aegypti* are mainly targeted with pyrethroid insecticides, i.e. deltamethrin and permethrin, which have been used since the late 1980s in Cambodia [10–13]. In the late 1960s, Mouchet and Chastel [14] showed that *Ae. aegypti* was highly susceptible to dichlorodiphenyltrichloroethane (DDT), fenitrothion, malathion and diazinon insecticides, but resistant to dieldrin and gamma-hexachlorocyclohexane. Recently, *Aedes* resistance to temephos was investigated in several field studies [10, 15, 16]. In a study carried out in 2001 [15], an *Aedes* population from Phnom Penh was found to be resistant to the insecticide temephos tested at the World Health Organization (WHO) diagnostic dose, while a population from Kampong Cham province was susceptible. More recently, among seven *Ae. aegypti* populations from Phnom Penh and Kandal provinces, six were found to be resistant to temephos, with mortality rates ranging from 11 up to 89% [16, 17]. Finally, eight different urban and peri-urban *Ae. aegypti* populations were tested with temephos, deltamethrin and permethrin in 2016: all of the populations showed resistance to insecticides used for vector control in Cambodia [10].

In the present study, we tested 14 adulticides belonging to the carbamate, organochlorine, organophosphate and pyrethroid families, and three larvicides, belonging to the organophosphate, spinosyn, and entomopathogenic bacteria [*Bacillus thuringiensis* ser. *Israelensis* (Bti)] families, to determine one or more effective insecticides for vector control strategies adapted for use against *Ae. aegypti* in Phnom Penh.

## Methods

### Mosquito sampling

One population of *Ae. aegypti* (F1) was sampled in 2021 on the Institut Pasteur du Cambodge campus (11°34′48.763 N; 104°54′54.212 E; World Geodetic System 84) in Phnom Penh. A susceptible United States Department of Agriculture (USDA) strain of *Ae. aegypti* was used as the control to test the effectiveness of the 17 insecticides [18].

Larvae and pupae were reared following standard conditions (i.e. temperature 27 ± 1 °C, relative humidity 75 ± 25%, photoperiod 12-h:12-h day/night) and were fed daily with half a teaspoon of fish food until adult emergence. Adult *Aedes* were reared under the same environmental conditions and fed with 10% sucrose solution. Female *Ae. aegypti* were blood-fed on laboratory-reared mice twice a week for 20 min. F1 generation eggs were collected on white filter paper placed inside black cups half filled with water. Eggs were harvested daily and dried and stored in envelopes at a relative humidity of 50%. F1 eggs were then immersed in water to obtain either larvae or adults for the different adult and larval bioassays.

### Adult* A. aegypti* bioassays

Standard testing procedures for adult* A. aegypti* followed WHO guidelines [19]. Twenty-five females older than 3 days of age were used and tested in WHO test tubes.

Papers impregnated with specific concentrations of the insecticides were obtained from the Vector Control Research Unit at the University of Science, Penang, Malaysia. Papers impregnated with insecticides at the following concentrations were used for the bioassays [19]: the carbamates bendiocarb at 0.1% and propoxur at 0.1%; the organochlorines DDT at 4% and dieldrin at 0.4 and 4%; the organophosphates fenitrothion at 1%, malathion at 0.8% and pirimiphos-methyl at 0.2%; and the pyrethroids alpha-cypermethrin at 0.03%, bifenthrin at 0.2%, cyfluthrin at 0.15%, deltamethrin at 0.03%, etofenprox at 0.5%, lambda-cyhalothrin at 0.03% and permethrin at 0.25%. Two concentrations of dieldrin were used to distinguish between the susceptible, resistant heterozygous and resistant genotypes [19].

For both the Phnom Penh population and the USDA susceptible reference strain, four WHO tube tests, each with 25 adult females and one impregnated paper, were undertaken for each insecticide. Thus, a total of 100 mosquitoes were tested per strain for each insecticide, to which they were exposed for 1 h. Mortality was assessed after 24 h.

As controls for each insecticide family, four kits were used with pyrethroid, organochlorine and organophosphate–carbamate control papers (WHO insecticide impregnated papers; Vector Control Research Unit), following the same test protocol described above for adult* A. aegypti*.

### Larval *A. aegypti* bioassays

In accordance with WHO guidelines [19], late third-instar larvae of the F1 generation were used to determine the susceptibility of the mosquito larvae to the three larvicides: temephos (PESTANAL, analytical grade, 250 mg; Sigma-Aldrich, St Louis, MO); Bti (VectoBac strain AM65-52; Valent BioSciences, Thailand); and spinosad (Sigma-Aldrich). Larvae showing any abnormalities were removed from the experiment. To determine the lethal dose (LD) required to kill 50% (LD_50_) and 90% (LD_90_) of the larvae, seven different concentrations were tested for each larvicide [20]. For the bioassays, temephos was used at 0, 5, 10, 20, 30, 50, 100 and 200 µg/L, spinosad at 0, 1, 5, 10, 25, 50, 100 and 200 µg/L and Bti at 0, 50, 100, 200, 400, 600, 800 and 1000 µg/L.

The positive control comprised a USDA susceptible reference strain with insecticide [18], and the negative control larvae in water without insecticide. For each of the tested insecticide concentrations and controls, four replicates (four 400-mL plastic goblets containing 200 mL of insecticide solution) with 25 larvae per replicate were used.

### Data management and statistical analysis

For adults, mortality was determined 24 h post-exposure. For the larvae, LD_50_ and LD_90_ were obtained by plotting mortality using log probit analysis with R Core Team software [21]. The LD_50_ and LD_95_ results obtained for the field strains were then divided by the results obtained using the USDA strain to calculate the resistance ratio (RR) for each field population.

## Results

### Adult bioassays

All of the negative controls realized with the control impregnated papers (pyrethroid, organochlorine and organophosphate–carbamate control papers) showed 0% mortality after 24 h. For the positive controls, 100% mortality of the USDA strain was observed for all of the insecticides. For the field strain, high mortality rates were observed with the carbamate, organochlorine (except for DDT) and organophosphate insecticides, while low mortality rates were observed with all of the pyrethroid insecticides.

**Fig. 1:**
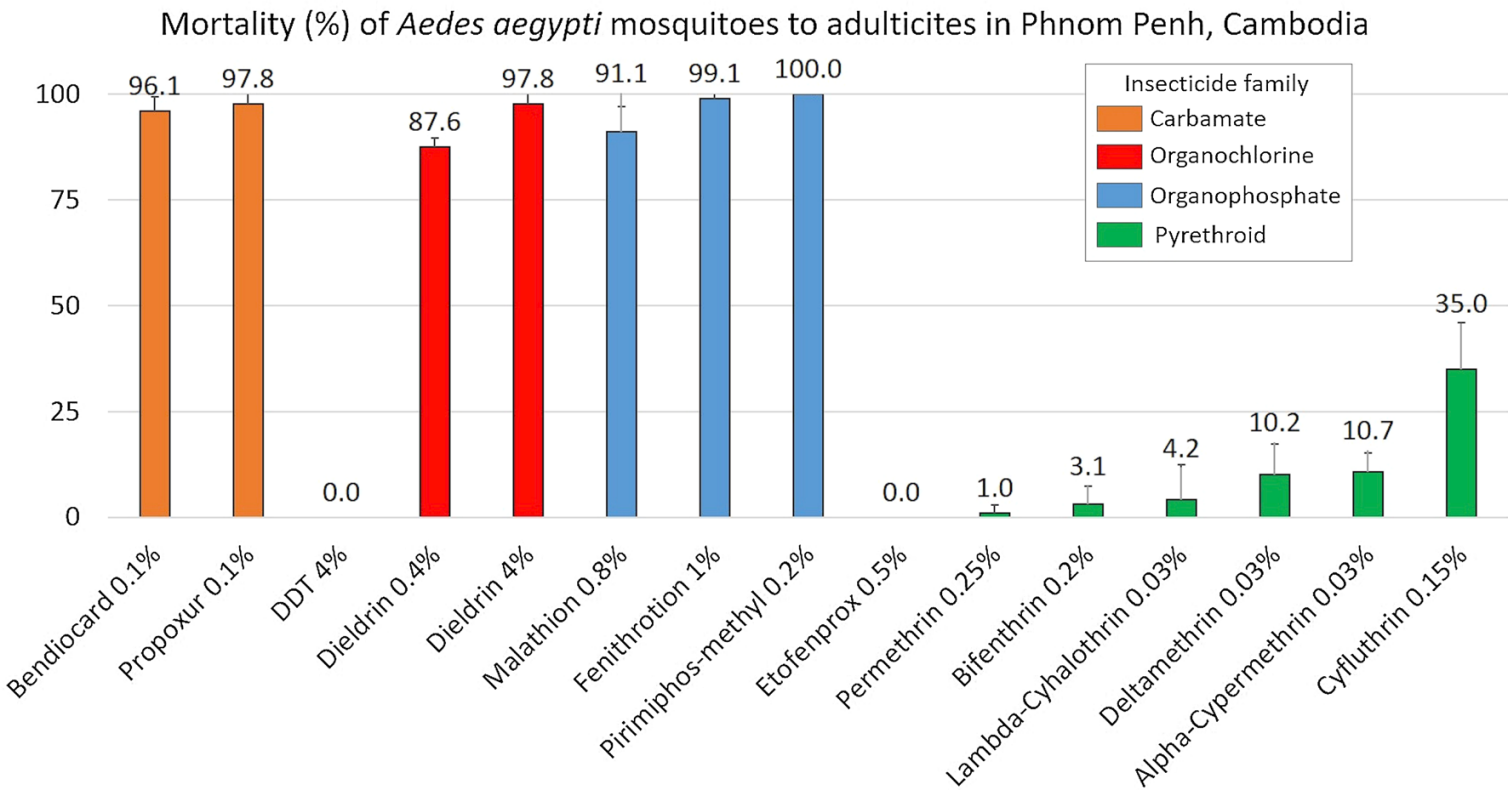
Mortality (%) of adult *Aedes aegypti* from Phnom Penh after exposure to the selected insecticides. *Error bars* indicate SDs

For the carbamates, adult *Ae. aegypti* mortalities were 96.1 ± 3.3% for 0.1% bendiocarb and 97.8 ± 2.6% for 0.1% propoxur. For the organochlorines, the mortality rates were 0% for 4% DDT and 87.6 ± 2.1% and 97.8 ± 4.3%, respectively, for 0.4% and 4% dieldrin. For the organophosphate insecticides, the adult mortalities were 91.1 ± 5.9% for 0.8% malathion, 99.1 ± 1.9% for 1% fenitrothion and 100% for 0.2% pirimiphos-methyl. For the pyrethroid insecticides, the adult mortalities were 0% for 0.5% etofenprox, 1 ± 1.9% for 0.25% permethrin, 3.1 ± 4.1% for 0.2% bifenthrin, 4.2 ± 8.3% for 0.03% lambda-cyhalothrin, 10.2 ± 7.1% for 0.03% deltamethrin, 11 ± 4.5% for 0.03% alpha-cypermethrin and 35 ± 11% for 0.15% cyfluthrin (Fig. 1).

### Larval bioassays

The LD_50_ for larvae of the USDA strain was 4.3 ± 0.2 µg/L for temephos, 55.2 ± 2.4 µg/L for spinosad and 4.4 ± 0.1 mg/L for Bti. The LD_90_ was 8.2 ± 0.5 µg/L for temephos, 72.3 ± 4.5 µg/L for spinosad and 4.9 ± 0.02 mg/L for Bti.

**Fig. 2:**
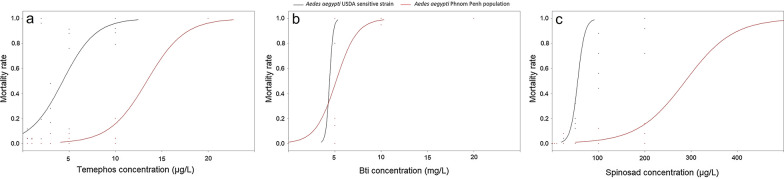
Mortality rate of *Aedes aegypti* larvae from Phnom Penh exposed to **a** spinosad (µg/L), **b** temephos (µg/L), and **c** *Bacillus thuringiensis* ser. *Israelensis* (*Bti*) (mg/L).* Black lines* Susceptible reference strain,* red lines* field population

For larvae of the Phnom Penh population of *Ae. aegypti*, the LD_50_ and LD_90_ for temephos were 13.6 ± 0.7 µg/L and 17.9 ± 0.8 µg/L, respectively, representing an RR_50_ of 3.1 and an RR_90_ of 2.2 (Table 1). For spinosad, the LD_50_ and LD_90_ were 287.8 ± 29.7 µg/L and 401.2 ± 49.9 µg/L, respectively, representing the highest RRs, i.e. an RR_50_ of 5.2 and an RR_90_ of 5.6. The lowest RRs were obtained with Bti (RR_50_ of 1.2 and RR_90_ of 1.6; LD_50_ of 5.2 ± 0.4 mg/L and LD_90_ of 7.6 ± 0.6 mg/L) (Fig. 2).

**Table 1:** Mean larval Lethal Dose (LD_50_ and LD_90_) ± SE of Phnom Penh population of Aedes aegypti with temephos, spinosad and Bti

Insecticides	USDA		Phnom Penh			
	LD_50_	LD_90_	LD_50_	RR_50_	LD_90_	RR_90_
Temephos (µg/L)	4.3 ± 0.2 (3.9–4.7), *n* = 809	8.2 ± 0.5 (7.2–9.2), *n* = 809	13.6 ± 0.7 (12.2–15.0), *n* = 798	3.1	17.9 ± 0.8 (16.3–19.5), *n* = 798	2.2
Spinosad (µg/L)	55.2 ± 2.4 (50.4–59.9), *n* = 746	72.3 ± 4.5 (63.4–81.1), *n* = 746	287.8 ± 29.7 (277.6–298.0), *n* = 750	5.2	401.2 ± 49.9 (303.3–499.1), *n* = 750	5.6
Bti (mg/L)	4.4 ± 0.1 (4.2–4.6), *n* = 807	4.9 ± 0.02 (4.8–5.0), *n* = 807	5.2 ± 0.4 2.8–7.6), *n* = 709	1.2	7.6 ± 0.6 (6.4–8.8), *n* = 709	1.6

*LD* Lethal Dose, *RR* Resistant Ratio, *SE* Standard Error. Between brackets, the 95% Confidence Interval. N is the number of larvae used in the experiment

## Discussion

In Cambodia, the main insecticides used to control mosquitoes are temephos, which is used against larvae, and permethrin and deltamethrin, which are used against adults. This study, like many previous ones [10, 14–16], demonstrated the resistance of *Ae. aegypti* to these insecticides. As the vector control of widely distributed species of mosquitoes in Cambodia is mainly based on the use of the larvicide temephos (235 t were imported into the country in 2020, according to a MoH report), we decided to assess the susceptibility of larvae of the Phnom Penh population tested here to two other larvicides that are also used worldwide, spinosad and Bti.

For spinosad, we observed a moderate resistance. Resistance to spinosad was also observed in an *Ae. aegypti* population in Lao People’s Democratic Republic (PDR) that was also resistant to temephos [20]. Furthermore, in Brazil, similar results were also obtained for spinosad, with mortality of larvae exceeding 80% and an RR_50_ ranging from 2.5 to 4.1 for populations that were highly resistant to temephos (for temephos, the RR_50_ ranged from 6.5 to 89.8 for all but one of the tested populations) [22]. The insecticide spinosad disrupts nicotinic acetylcholine receptors, which leads to paralysis, followed by death. The mechanism of resistance that has thus far been described is a mutation of the beta subunit of a gene coding for a nicotinic acetylcholine receptor [23]. It would be interesting to determine if the Phnom Penh population has a mutation within the same area of its genome, or to know if this phenotypic resistance is due to another selected mechanism (i.e. cross-resistance) with temephos (i.e. enzymatic detoxification).

The* Ae. aegypti* population was susceptible to Bti. This result agrees with those previously reported for *Ae. aegypti* in Cambodia, when Bti was successfully tested around Phnom Penh in 2005 and 2016 [16, 17]. Furthermore, in Lao PDR, promising results were found for Bti against wild temephos-resistant populations of *Ae. aegypti* in the laboratory and in semi-field trials [20]. In light of these results, Lao PDR MoH changed its policy and decided to use Bti instead of temephos in 2019. Bti appears to be a good alternative to temephos for use as a larvicide in Cambodia.

One of the most important findings of our study is the very low mortality, and therefore high resistance, of the *Ae. aegypti* adults to all the tested pyrethroids, including the two routinely used in Cambodia, permethrin and deltamethrin. Resistance to these two pyrethroids has been previously described, and their ineffectiveness recognized [10, 24]. The two most widely recognized mechanisms of insecticide resistance are voltage-gated channel modification (knockdown resistance mutation; *kdr*) and the overproduction of detoxification enzymes [25, 26]. Even though pyrethroids target sodium channels [27], resistance mechanisms are not specific to this particular insecticide family but to the structural conformation of each pyrethroid insecticide. This might explain why the same phenotypic pattern of resistance is rarely observed between type I and II pyrethroid forms and pseudo-pyrethroids that are non-ester pyrethroids, e.g. etofenprox [28]. Observed resistance to all pyrethroids as well as to the organochlorine DDT suggests that several *kdr* mutations have been selected as a result of mosquitoes coming into repeated contact over decades with different insecticides that synergize when combined [29, 30]. Indeed, very high levels of pyrethroid resistance have been reported, with some individuals carrying two (S989P + V1016G) or three mutations (S989P + V1016G + F1534C) in their sodium channel genes [31, 32]. The description of several *kdr*-associated mutations seems to validate this hypothesis [27, 31, 33]. Studies have shown the presence of different *kdr* mutations in Southeast Asia [29, 34, 35]. Hence, the use of pyrethroids in Cambodia, Lao PDR [20] and Thailand [32, 36] is clearly compromised.

Furthermore, although organochlorine and organophosphate insecticides, with the exception of DDT, cause high mortality in adult *Ae. aegypti*, they cannot be recommended for vector control due to their broad spectrum of action and persistence in the environment. Fortunately, the two tested carbamates, bendiocarb and propoxur, were effective at killing the mosquitoes. In an Australian study [37] carried out in 1999, *Ae. aegypti* was found to be resistant to bendiocarb, while there was no evidence that it was resistant to pyrethroids. A decrease in susceptibility of this species to bendiocarb was also observed in Colombia [38], Malaysia [39], Trinidad and Tobago [40], and Mexico [41]. However, no resistance to bendiocarb was detected in this species in Costa Rica [42] or in a study carried out in Mexico [43]. *Ae. aegypti* showed no resistance to propoxur in studies carried out in Australia [37], Colombia [44], Mexico [43] and Panama [45]. Resistance to propoxur was found in three of the ten tested *Ae. aegypti* populations in a study undertaken in Colombia [38], and was more frequent in *Ae. aegypti* in Malaysia [39]. In sum, bendiocarb and propoxur are considered good potential alternative insecticides for the control of *Ae. aegypti* during outbreak events in Cambodia.

## Conclusions

At least two conclusions have emerged from this work: further study of insecticide resistance, a major concern in Cambodia, should be a public health priority for the country; alternative and innovative vector control strategies should be developed for Cambodia. The study of insecticide resistance, including a focus on molecular bases and biomarkers, which is presently not an axis of research in Cambodia, should be implemented as soon as possible to help orientate the MoH in its fight against vector species of mosquito. Recently developed effective vector control strategies which use *Wolbachia* against *Ae. aegypti* could be a promising tool for use in Cambodia and throughout Southeast Asia. Finally, Bti should be considered a very good candidate alternative insecticide to temephos for the control of *Ae. aegypti* larvae, and the adulticides propoxur and bendiocarb (carbamate) for the control of *Ae. aegypti* on land under mosaic farming or crop rotation.

## Data Availability

All data generated or analyzed during this study are included in this published article and its additional files.
